# We Are All in This Together: Effects of Synchrony in Social Media Videos on Viewers’ Experience of Self-Transcendent Emotions

**DOI:** 10.3390/bs16071155

**Published:** 2026-07-09

**Authors:** Mary Beth Oliver, Alex Paloma, Yansheng Liu, Yilan Guo, Jack Waier, Hannah Xiangruo Huang, Katherine Ryan

**Affiliations:** Department of Media Studies, Donald P. Bellisario College of Communications, Penn State University, University Park, PA 16802, USA; atp5473@psu.edu (A.P.); yvl6116@psu.edu (Y.L.); ybg5203@psu.edu (Y.G.); jjw7059@psu.edu (J.W.); xjh5145@psu.edu (H.X.H.); katherineryan@psu.edu (K.R.)

**Keywords:** positive media psychology, self-transcendent affect, social media, synchrony

## Abstract

Synchrony refers to similarity in movement between different actors such as dancing, clapping, or singing together. Prior research demonstrates that synchrony often elicits emotions that may be characterized as aesthetic or self-transcendent (e.g., awe, connectedness). Our research situated the concept of synchrony in media contexts, examining viewers’ affective responses to videos featuring synchronous movement. Study 1 employed an experiment, showing that synchronized videos elicited greater awe, with awe associated with a host of prosocial outcomes reflecting connectedness and motivations to do good. Study 2 employed content analytic procedures to examine how the synchrony present in a large sample of YouTube videos was associated with user comments reflecting self-transcendent emotions, and how these emotions were associated with the salience of moral foundations. The results showed that synchrony was associated with greater feelings of awe, admiration, and elevation. Further, comments reflecting self-transcendence were associated with the salience of moral foundations, and particularly the foundation of care.

## 1. Introduction

Our delight in synchrony is evidenced in many settings, ranging from tapping our feet in rhythm to a musical performance, practicing tai-chi in a tranquil city park, or feeling mesmerized by a murmur of starlings as they dance in breath-like movements in a twilight sky. Synchrony appears to touch us deeply, engage our imaginations, and elicit wonder and appreciation.

Synchrony is not only evident in nature and in social gatherings but is also present in many of our social media offerings. YouTube videos of flash mobs (Wang et al., 2014) are abundant, with viewers delighting in the seemingly spontaneous and joyous displays of collective dancing and singing. Likewise, TikTok dance crazes not only feature people dancing with one another, but also include other users joining in the fun by sharing their own renditions in locations throughout the world (Griffiths, 2023). Although some critics may characterize these contemporary media offerings as silly diversions, others argue that they provide users with a sense of wonder and an opportunity for connection (e.g., Thau, 2023).

The effects of synchrony on individuals’ affective responses and feelings of affiliation have long been the focus of scholarly research (Valdesolo & DeSteno, 2011). However, this research has typically focused on actual, “real life” interactions. In that context, synchrony often results in greater feelings of affiliation and in emotional responses that may be characterized as aesthetic or self-transcendent (e.g., awe, connectedness; see Chartrand & Lakin, 2013). Yet synchrony abounds in other contexts as well, with social media being a widely viewed and shared experience. Consequently, this research builds upon existing work in positive media psychology to examine viewers’ affective responses to videos featured on social media, seeking to explore if media synchrony can elicit self-transcendent emotions that are also associated with prosocial outcomes.

### 1.1. Positive Media Psychology

Over the past decade, researchers have increasingly explored the positive and uplifting aspects of media, focusing on how they can inspire individuals, encourage personal growth, reveal the compassionate side of human nature, and foster a sense of connection with diverse communities (Oliver et al., 2021; Raney et al., 2021). In earlier studies, Oliver and Raney (2011) drew from Aristotle’s concept of well-being, distinguishing between hedonic well-being (associated with pleasure) and eudaimonic well-being (related to human flourishing). Eudaimonia encompasses experiences that may not necessarily be pleasurable but are deeply meaningful, such as personal development, insight into life’s purpose, and self-realization.

Over time, as research in positive media psychology continued to attract attention, scholarship began to broaden to include a wider range of user responses. This shift involved moving beyond solely studying eudaimonic gratifications to examining how media can trigger self-transcendent reactions (Oliver et al., 2018). In contrast to eudaimonic experiences, self-transcendent responses involve shifting attention away from personal concerns toward a broader awareness of others, nature, or even the cosmos. This outward shift can lead to a deeper sense of interconnectedness, helping individuals recognize that despite differences in culture, nationality, or life experiences, we share common human aspirations, dreams, and challenges (Yaden et al., 2017).

There are various forms of self-transcendent responses, with some receiving more academic attention than others. One well-studied example is elevation, an emotional response that occurs when witnessing acts of “moral beauty” such as kindness, compassion, or generosity. Experiencing elevation can result in physical sensations like tears, a lump in the throat, or chills (Algoe & Haidt, 2009). Another related concept is kama muta, derived from Sanskrit, which describes the feeling of being emotionally moved by communal sharing, like family members reuniting after a long separation (Fiske et al., 2017). Additionally, recent studies have focused on the emotion of awe, which can be sparked by vast landscapes, starry skies, or a sweeping rainbow. Awe typically involves a sense of smallness in the face of something immense, requires a shift in perspective to process the magnitude of the experience, and involves a sense of connection with others and/or the universe at large (Keltner & Haidt, 2003). Because there are a variety of self-transcendent emotions, in this research we commonly use the phrases “self-transcendence” and “self-transcendent emotions” interchangeably.

Researchers have also begun to examine the specific types of media platforms and media portrayals that elicit feelings of self-transcendence. In general, this work suggests that people find inspiring content among a wide variety of media platforms. For example, two national studies of people in the U.S. found that the vast majority of people have experienced inspiration from media, including from music, film, books, television, and social media, among many others (Janicke-Bowles et al., 2021; Raney et al., 2018).

In terms of specific content, several content analyses give us insight into what may inspire users. For example, Dale et al. (2017) analyzed YouTube videos tagged as “inspirational” and found that the most typical portrayals depicted hope, appreciation of beauty and excellence, and gratitude. Similarly, Ji et al. (2019) examined inspiring versus non-inspiring New York Times news stories that were frequently shared. This study found that words related to awe, elevation, hope, and gratitude were more frequently included in inspirational than non-inspirational stories. Researchers have also expanded this research to include contemporary types of content that may evoke inspiration. For example, Rieger and Klimmt (2019) studied memes associated with the hashtags #inspirational and #meaningful to see what additional hashtags were present. Their findings mirrored those of previous work, with the target hashtags associated with hashtags such as #love, #beautiful, #happy, #peace, and #wisdom.

With this background in mind, the present study introduces a novel variable that is associated with many of the prosocial outcomes such as affiliation and connectedness that have been examined in extant research. Namely, we examine if displays of synchrony may result in heightened self-transcendent experiences and the prosocial outcomes that often follow. Because this is a new direction, in the next section we overview research on synchrony per se and then turn to how this may inform media research.

### 1.2. Research on Synchrony

Synchrony is generally thought to reflect bodily movements or physiological states that are temporally mirrored or shared among individuals or groups of individuals (daSilva & Wood, 2024). Although these movements may reflect practiced coordination such as dancing or playing music, they frequently occur spontaneously, such as nodding one’s head or leaning forward during a conversation.

Interest in synchrony has dramatically increased over the last decade. daSilva and Wood (2024) suggested that part of this research attention reflects advances in measurement and statistical techniques that allow for analysis of continuous movements that are often very subtle, if not imperceptible. Additionally, though, interest in synchrony likely reflects the host of prosocial outcomes that arise from synchronous interaction.

In a recent meta-analysis of interpersonal synchrony in 43 independent studies, Mogan et al. (2017) examined four positive outcomes associated with synchrony: prosocial behavior, positive affect, social cognition, and perceived social bonding. Many other scholars have reported similar outcomes, with some reporting affective responses that are akin to self-transcendent emotions (e.g., awe, elevation) that are frequently examined by media psychologists (Wlodarczyk et al., 2023; Wlodarczyk et al., 2020).

The explanations for the connection between synchrony and positive outcomes are diverse, ranging from evolutionary to social ritual to neurological. For example, some scholars have suggested that synchrony helps in building and maintaining social bonds (Rennung & Göritz, 2016). Other scholars have suggested that community events that involve synchrony, such as rituals, reading passages, or singing, help to strengthen community ties (Páez et al., 2015). Given the diversity of contexts in which synchrony plays important roles in heightening affiliation, we believe that it may operate in similar ways in the context of media portrayals.

### 1.3. Synchronous Displays in Media

Research on synchrony has primarily been studied in face-to-face interpersonal or group contexts, including between friends, parents and children, strangers, or groups. Yet recent scholarship has begun to explore synchrony in mediated contexts. For example, Stupacher et al. (2022) utilized EEG scans to assess neural synchrony between mother–teen dyads as they talked face-to-face or texted from different rooms. These authors found that both talking and texting were associated with heightened synchrony.

Although texting and video chats are certainly forms of mediated communication, in this study we are interested in how simply viewing synchrony may elicit emotions such as awe and elevation that have been studied frequently by media scholars (Raney et al., 2021). Part of the motivation for this focus is anecdotal—examples of synchronous behaviors abound in social media, including the aforementioned examples of flash mobs, TikTok videos, musical performances, and even nature videos. Additionally, there is some evidence that simply imagining synchrony with others can heighten affiliation (Crossey et al., 2021). Finally, media portrayals afford the potential to observe synchrony in a wide variety of locations and cultures, thereby opening a potential route to heighten feelings of connectedness and affiliation with geographically diverse audiences.

In this research, we employed a very broad approach to conceptualizing synchrony. Namely, our focus was on behavioral similarity in movement. We note that other scholars have offered more narrow or specific definitions to include additional elements such as a shared focus of attention and the experience of arousing and positive emotions (Wlodarczyk et al., 2020). Although we believe that these additional elements add depth and nuance to the concept, we opted to employ a broad approach so that we could explore potential moderators of the relationship between synchrony and feelings of self-transcendence that it may elicit.

With this background in mind, we conducted two studies to explore media synchrony and viewer experience. The first study involved experimental methods, and the second involved content analytic procedures examining YouTube videos varying in synchrony and their associated user comments. For both studies, our research was broadly interested in three related questions:

RQ1: Do media portrayals featuring synchrony elicit self-transcendent emotions (e.g., elevation, awe)?

RQ2: Do media portrayals featuring synchrony have indirect effects on prosocial outcomes, mediated by self-transcendent emotions?

In addition to examining the effects of synchrony on self-transcendent emotions, we were also interested in examining additional aspects of the videos that may moderate this relationship. For example, we reasoned that voluntary involvement, friendly interactions, and positive affect among the participants in the videos may serve to increase the association of the synchrony–transcendence relationship, whereas poor video quality, anger, or violence may result in weaker or even negative relationships. For example, soldiers marching at the behest of a dictatorial leader may feature synchrony, but their involuntary participation and their potential fear of punishment for non-compliance may dampen viewers’ self-transcendence because the synchrony is forced and is devoid of displays of positive affect. Because the possible moderators are wide-ranging, in Study 2 we examined a host of variables that we reasoned may be consequential.

RQ3: What aspects of media heighten or dampen the effects of synchrony on viewers’ responses?

## 2. Study 1

### 2.1. Method

#### 2.1.1. Design and Procedures

This study employed a 2 (Synchronized: Lower, Higher) × 3 (Focus: Humans, Animals, Objects) online experimental design. Two videos were employed to represent each experimental condition, with participants viewing only one video. During the study, participants first answered some basic demographic questions. They were then presented with a short video to verify that they could both see and hear videos on the online platform. Subsequently, participants were assigned to view one of the 12 videos employed. They then provided their responses to the video, including how much they were perceived as elevating and awe-inspiring. They then completed several sets of questions pertaining to down-stream effects, including universal orientation, prosocial motivations, and connectedness to other humans and to the planet in general.

#### 2.1.2. Participants

Participants were recruited from CloudResearch and were offered $0.75 USD for their participation with a $0.25 bonus upon completion of the questionnaire. Originally, 255 participants were recruited. Three people stopped their participation prior to viewing the video, 2 people spent an inordinate amount of time on the questionnaire (>3 *SD* above the mean), and 18 cases were identified as duplicates. After removing these problematic cases, the final sample included 232 participants. These participants were predominantly (58.2%) women (men = 39.7%; trans/non-conforming/preferred not to answer = 2.1%), had a median age of 41 years, and self-identified as White (79.7%; Black = 10.3%; Asian = 7.3%). Approximately half of the sample (55.2%) had completed a 4-year college degree.

#### 2.1.3. Measures

To assess emotional responses, participants were presented with a series of adjectives and were asked to indicate the extent to which each adjective was descriptive of the video using scales ranging from 1 (*Not at All*) to 7 (*Very Much*). Three variables were employed to represent awe (awe-inspiring, breathtaking, beautiful; *M* = 4.54, *SD* = 1.57, Cronbach’s α = .90) and three to represent elevation (moving, touching, meaningful; *M* = 4.52, *SD* = 1.62, Cronbach’s α = .88). We also assessed overall evaluation of the video so as to rule out the possibility that different ratings of elevation and awe simply reflected more favorable responses to synchronized versus non-synchronized videos (e.g., interesting, unpleasant(r), boring(r); *M* = 5.82, *SD* = 1.16, Cronbach’s α = .71).

Universal orientation was assessed using six items taken from Piedmont’s (1999) universality scale, Phillips and Ziller’s (1997) universal orientation scale, and Aquino et al.’s (2011) views of humanity scale (e.g., “All life is interconnected”; *M* = 4.42, *SD* = 1.57, Cronbach’s α = .94). Prosocial motivations were assessed using three items reported in the study by Algoe and Haidt (2009; e.g., “Being a better person”; *M* = 5.35, *SD* = 1.22, Cronbach’s α = .92). Finally, two additional single-item measures were created using a variation of Aron et al.’s (1992) inclusion of others in the self that reflects closeness with others on scales ranging from 1 to 100. In our study, we assessed feelings of closeness with people throughout the world (*M* = 38.21, *SD* = 27.74) and with all living things throughout the world (*M* = 44.80, *SD* = 31.32).

#### 2.1.4. Stimuli

The selection of videos was based on a pretest of 24 videos. From those 24 videos, two were selected to represent people, two to represent animals, and two to represent objects. One of the synchronized videos of people included a large group of people playing and singing “Have You Ever Seen the Rain?” and the other included street musicians throughout the world singing “What’s Going On?” The non-synchronized versions featured single artists singing these same songs. For the videos featuring animals, both synchronized and non-synchronized videos included fish swimming and sheep in pastures. For the videos featuring objects, both synchronized and non-synchronized videos featured colorful balls and public fountains. Appendix A provides greater detail about the pretest.

In this study, as in the pretest, perceived synchrony was assessed on 7-point scales using seven items adapted from Bernieri (1988) (e.g., coordinated movement, behavioral similarity; *M* = 5.50, *SD* = 1.03, Cronbach’s α = 0.87). As a manipulation check, we conducted a 2 (Synchronized: Lower, Higher) × 3 (Focus: Humans, Animals, Objects) analysis of variance (ANOVA) on ratings of perceived synchrony. This analysis revealed only a main effect of Synchrony, with participants reporting greater perceived synchrony in the higher-synchronized condition (*M* = 5.90, *SE* = 0.09) than in the lower-synchronized condition (*M* = 5.14, *SE* = 0.09), *F*(1, 226) = 33.00, *p* < 0.001, η_p_^2^ = 0.14.

### 2.2. Results

The first analysis examined whether videos that display synchrony elicit greater feelings of elevation and awe than videos without synchrony. Because ratings of overall evaluation were positively associated with both awe (*r* = 0.75, *p* < 0.001) and elevation (*r* = 0.52, *p* < 0.001), we opted to use evaluation ratings as a covariate. Specifically, a 2 (Synchronized: Lower, Higher) × 3 (Focus: Humans, Animals, Objects) multivariate analysis of covariance (MANCOVA) was conducted on ratings of awe and elevation, controlling for overall evaluation. The multivariate effects revealed significant main effects for both synchrony, Wilks’ λ = 0.97, *F*(2, 224) = 3.54, *p* = 0.031, η_p_^2^ = 0.03, and focus, Wilks’ λ = 0.75, *F*(4, 448) = 17.42, *p* < 0.001, η_p_^2^ = 0.13. No significant interactions were observed.

An examination of the univariate results for ratings of elevation revealed only a main effect of focus, *F*(1, 225) = 27.88, *p* < 0.001, η_p_^2^ = 0.20. Post hoc pairwise comparisons using least significant difference (LSD) tests (O’Keefe, 2003) showed that videos featuring humans were rated as significantly more elevating (*M* = 5.33, *SE* = 0.14) than videos featuring animals (*M* = 4.34, *SE* = 0.14) or objects (*M* = 3.85, *SE* = 0.14).

In contrast, univariate results for ratings of awe revealed main effects for both focus, *F*(2, 226) = 6.05, *p* = 0.003, η_p_^2^ = 0.05, and synchrony, *F*(1, 226) = 5.23, *p* = 0.023, η_p_^2^ = 0.02. Awe was rated higher for videos featuring humans (*M* = 4.76, *SE* = 0.14) or animals (*M* = 4.74, *SE* = 0.14) than videos featuring objects (*M* = 4.15, *SE* = 0.14). Importantly, ratings of awe were higher for the higher-synchronization videos (*M* = 4.74, *SE* = 0.12) than for lower- synchronization videos (*M* = 4.36, *SE* = 0.12).

The second analysis pertained to downstream effects regarding universal orientation, prosocial motivations, and feelings of connectedness. This analysis used the manipulated synchrony videos as the IV and awe as the mediator. We opted not to examine elevation as a mediating variable, as we found no evidence that our manipulation of synchrony had any appreciable effect on this variable. We also opted not to include the focus of the video in this analysis because, although we found main effects of focus on awe, there was no Synchrony X Focus interaction. However, the focus of the video (dummy-coded) was employed as a covariate.

**Figure 1:**
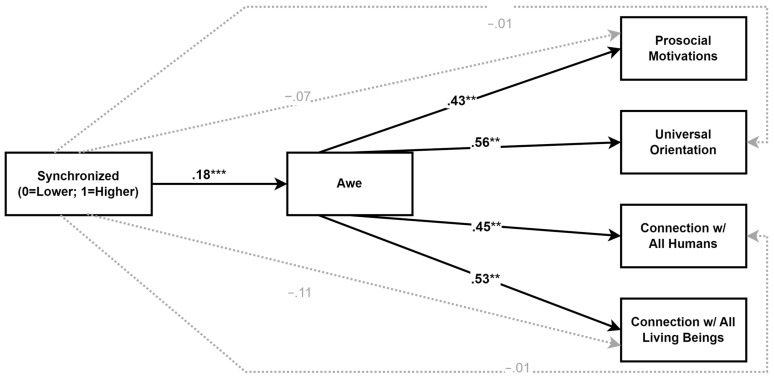
Study 1: Mediation Analysis of Indirect Effects of Synchrony. ** *p* < 0.01. *** *p* < 0.001. *Note*. Path weights are standardized coefficients.

Figure 1 illustrates the results of this path analysis. As this figure shows, higher levels of synchronization were a significant predictor of heightened awe (as demonstrated, too, in our analysis of variance). Further, awe was positively predictive of prosocial motivations and universal orientation, and heightened feelings of connection with humanity and all living beings. Additionally, the indirect effects of synchrony on each of these outcomes was significant: prosocial motivations (95% CI: [0.026, 0.137]); universal orientation (95% CI: [0.033, 0.173]); connection with humanity (95% CI: [0.027, 0.147]); connection with all living beings (95% CI: [0.024, 0.162]).

### 2.3. Brief Discussion

This study shows that synchrony in videos can result in heightened self-transcendent emotion, but only for awe and not for elevation. In many respects, this finding may not be surprising, as elevation is thought to result from viewing exemplary displays of moral beauty or virtue. In our case, the videos did not display any particular virtue, but rather focused on similarity of movement. We also found that the topic of the video was consequential. Namely, ratings of awe were higher for videos featuring animals or humans rather than inanimate objects. Importantly, mediation analyses showed indirect effects of synchrony on all of our measures of prosocial outcomes.

Based on this initial test, we feel confident in arguing that viewing synchrony can lead to self-transcendence and associated prosocial outcomes *under some circumstances*. Namely, this study found effects only for awe and particularly for videos featuring animals or humans. Beyond this, it is somewhat difficult to make any broader generalizations, as our stimuli were limited in scope and we took efforts to hold other variables as constant as possible given this experimental context.

To expand upon these findings, we opted to conduct a second study to examine a broader array of videos and to explore user responses in a different context. More specifically, we turned our attention to existing videos posted on YouTube and the user comments that these videos elicited.

## 3. Study 2

The purpose of this study was to expand the sample of videos to not only broaden the wealth of videos that are available to viewers, but also to use more “natural” responses to the videos as reflected in user comments. With a larger sample of videos, we also aimed to examine additional aspects of the videos that may heighten or dampen the relationship between synchrony and user comments reflecting the experience of self-transcendent emotions.

For each video in our sample, we collected meta-data (e.g., number of views) and additional characteristics, including ratings of the amount of synchrony featured in the video. Finally, we then examined user comments and assessed the extent to which they reflected self-transcendent affect and the salience of moral dimensions (e.g., care, fairness). Ultimately, each video was treated as a unit of analysis, with aspects of the videos (i.e., synchrony) used to predict comments reflecting self-transcendence, and self-transcendence used to predict the salience of moral dimensions. Below we describe how the sample of videos was gathered, how ratings of the videos were assessed, and how we coded user comments. A more elaborate discussion of our methods and validation tests can be found in Appendix B, Appendix C and Appendix D.

### 3.1. Method

#### 3.1.1. Sample of YouTube Videos

Prior to collecting a sample of videos, we first examined the literature on synchrony to collect scenarios or contexts frequently mentioned as illustrating or involving synchronous behaviors. These scenarios included marching, dancing, clapping, drumming, religious and cultural rituals, parades, political demonstrations, birds in flocks, and schools of fish, among many other topics (see, for example, Bouchat et al., 2024; Edelman & Harring, 2015; Gordon et al., 2020; Monroy et al., 2021; Rennung & Göritz, 2016). Based on this literature, we created five broad categories of topics: (a) dancing/dance performance, (b) drumming/clapping, (c) nature, (d) parades/marching/walking, and (e) rituals.

With these topics in mind, in April of 2026 we provided prompts to Perplexity AI (Perplexity Computer, Cloud-Native Agent) (https://www.perplexity.ai/, accessed on 30 June 2026) to generate a sample of 100 YouTube URLs for each topic, with half of the videos in each topic stressing synchronous interactions and the others not stressing synchronous interactions. We presented a total of 10 different prompts, one for each topic/synchronization instructions. Our prompts instructed that the duration of the videos could range between 20 and 420 s, that each video should have at least 10 user comments, that there should be no duplicates, and that no more than two videos should come from the same channel. For each prompt, we asked that 50 URLs be generated. Appendix B provides the specific prompts that we employed to generate our sample of videos.

After the URLs were generated, our team divided up the URLs to view and to note any problematic videos. The first author reviewed these notes. After removing redundant videos (e.g., different videos of the same parade, *n* = 5), videos that included no display of the topics of interest (*n* = 4), and videos that were unavailable or private (*n* = 2), our final sample included 489 videos. The median values for aspects of these videos include a duration of 226 s, a view count of 311,391, a like count of 2870, and a comment count of 200. A full list of the URLs examined can be found at https://osf.io/nz42a/overview?view_only=0adb736ced7840b0a8d4d749b5aa3314 (accessed on 30 June 2026).

It is important to note that our identification of the topic categories was meant to create a diversity of videos on scenarios relevant to synchrony, but that could be asynchronous as well. Obtaining variance in the level of synchrony was a primary goal so that we could examine the extent to which the level of synchrony displayed corresponded to user comments. However, because our prompts were broad, we did not assume our prompts regarding synchrony would result in clearly synchronous versus non-synchronous videos. For example, a video showing people dancing at a concert may feature a wealth of asynchronous movement, but some movements (e.g., clapping) may be synchronous as well. As a result, our prompts and the resultant videos were subjected to further coding to more clearly identify both the level of synchrony displayed as well as additional characteristics about the videos that we reasoned might play a role in heightening or damping the effect of synchrony on users’ experience of self-transcendent emotion.

#### 3.1.2. Coding of Video Characteristics

To collect additional information concerning characteristics of these videos, we used Viewpoints AI, an AI-based research platform for designing and running studies with simulated personas. These are powered by underlying LLMs, which are configured as part of the study. In the current study, the underlying model used was Gemini-2.5-flash. The simulated personas on this platform can view images/video/stimuli and can respond using a variety of question types (e.g., Likert measures, open-ended questions, ranking, etc.). Further, parameters of the synthetic personas can be specified to reflect desired demographic characteristics (e.g., gender, age). In this study, we specified approximately equal numbers of males and females that could range in age from 18 to 76 years. In this study, five unique AI agents viewed and rated each video. We averaged the ratings across the five agents to create a single score for each item measured.

Before beginning our study, we conducted a validity test using the same data and videos used in Study 1. Appendix C describes how we compared data from Study 1 with responses from AI Agents on the Viewpoints AI platform. Because this validity test led us to be reasonably confident that the AI agents could adequately view and rate videos, we proceeded to craft the ratings used in this study. First, we employed the same synchrony ratings that we employed in Study 1. In this study, the resulting synchrony ratings from AI agents had a strong level of reliability (Cronbach’s α = 0.93). Second, we added additional items for aspects of the videos that we reasoned may heighten or moderate the effect of synchrony, including perceptions that the participants in the video wanted to participate, that they enjoyed the experience, that they liked one another, and that the behaviors were natural and spontaneous. We also reasoned that group density, size, and diversity may also heighten the effects of synchrony, as these characteristics may encourage perceptions of inclusion. Finally, we also considered aspects of the video that may dampen self-transcendent responses, including displays of violence or anger, the inclusion of the content as part of a news story, as well as poor video quality. Each of these potential moderators was measured on single-item scales ranging from 1 (*Not at All*) to 7 (*Very Much*). Appendix D describes how we tested the validity of these additional measures using a subset of videos (*n* = 10) that were rated by both humans and AI agents.

#### 3.1.3. Collection and Analysis of User Comments

To examine how users respond to videos with varying levels of synchrony, we use Python 3.14 and YouTube’s data API v3. More specifically, we retrieved up to 200 top-level comments (not replies) per video. If a video had more than 200 top-level comments, we retrieved 200 comments with the most likes. We also had posts in languages other than English translated as part of the retrieval process. The Python code that was employed in scraping the user comments can be found at https://osf.io/nz42a/overview?view_only=0adb736ced7840b0a8d4d749b5aa3314 (accessed on 30 June 2026).

To analyze user comments, we employed WordStat v. 2026.1 (Provalis Research, 2026). WordStat is a text mining and content analysis software that allows users to observe word frequencies and topics, and that also allows users to develop their own dictionaries on topics of interest. In our case, we employed two dictionaries. First, we used Ji et al.’s (2019) Self-Transcendent Emotion Dictionary (STED). This validated dictionary codes for words indicative of five different self-transcendent emotions: elevation, awe, admiration, gratitude, and hope. Because the videos in our sample had varying numbers of comments, counting the raw number of words overall would have given more weight to videos with more comments. Hence, we recorded the % of cases (or individual comments) for each video that included terms for each self-transcendent emotion in the dictionary. Additionally, because there were words in the dictionary that were included in more than one emotion category (e.g., “*amazed*” was included in both the awe and the admiration categories), we also computed an overall self-transcendent measure that reflected the % of comments for each video that contained *any* of the self-transcendent terms.

To our knowledge, textual dictionaries for prosocial outcomes that mirror the ones we employed in Study 1 have not yet been developed. Consequently, we employed the Moral Foundations Dictionary 2.0 (Frimer et al., 2019) as a proxy. This cross-culturally validated dictionary is based on Haidt’s (2012) research on moral intuitions, identifying the most salient dimensions by which individuals make moral judgments. The dimensions included in this dictionary include care (e.g., kindness, compassion, empathy), fairness (e.g., equality, justice), loyalty (e.g., loyalty, fidelity), authority (e.g., respect, obey), and sanctity (e.g., purity, sacred). Further, this dictionary includes two valences for each foundation: virtue (words describing the presence of the foundation) and vice (words describing the absence of the foundation). In this study, we examined only the virtue valences. Our primary interest was in the foundation of care because we believe it most closely approximates the prosocial outcomes assessed in the first study, with words such as “empathy,” “helping,” and “generosity” being employed to signify this dimension. Although this dictionary likely captures the concept of prosocial motivations employed in Study 1, it is important to note that other concepts such as universal orientation and connectedness are not evident in the dictionary. We also opted to include all moral foundations to examine if synchrony predicts all foundations or if it appears to be more relevant to some dimensions than to others. The word dictionaries and the derived data from user comments can be found at https://osf.io/nz42a/overview?view_only=0adb736ced7840b0a8d4d749b5aa3314 (accessed on 30 June 2026).

### 3.2. Results

#### 3.2.1. Ratings of Synchrony

We first tested the validity of our measure of synchrony by conducting a 2 (Synchronized Specified in Search: Yes, No) × 5 (Topic Specified in Search: Dancing, Drumming/Clapping, Nature/Animals, Parades/Marching/Walking, Rituals/Ceremonies) analysis of co-variance (ANCOVA) on ratings of synchrony, controlling for the number of view counts, like counts, and comment counts. Of note, the only significant covariate was for comment counts, and hence, the other two covariates were not retained in further analyses. Table 1 displays the means, standard errors, and LSD post hoc comparisons for this analysis. First, a main effect was obtained for Synchronized Search, *F*(1, 459) = 165.84, *p* < 0.001, η_p_^2^ = 0.27, with higher scores obtained for the synchronized-search videos (*M* = 6.83, *SE* = 0.05) than for the videos where synchronization was not specified (*M* = 5.88, *SE* = 0.05). However, we also obtained a significant main effect for topic, *F*(4, 459) = 18.72, *p* < 0.001, η_p_^2^ = 0.14. As Table 1 shows, the search for nature/animals resulted in videos that were rated lower on synchrony than any other topic. Finally, a Synchronized Search × Topic Search interaction was also obtained, *F*(4, 459) = 9.89, *p* < 0.001, η_p_^2^ = 0.08. Table 1 shows that among the non-synchronized videos, nature videos scored the lowest and dancing and drumming/clapping videos the highest on synchrony. Among the synchronized videos, the scores were more similar, though nature videos scored lower than videos featuring dancing or parades/marching/walking. Importantly, though, for each topic category, ratings were higher for the synchronized-search videos than for the videos where synchronization was not specified.

#### 3.2.2. Synchrony and Comments Reflecting Self-Transcendent Emotion

We first tested whether synchrony scores were associated with comments reflecting self-transcendent emotion. Specifically, we ran partial correlations between synchrony and each of the individual self-transcendent affect scales as well as for the overall self-transcendence measure, controlling for the search topic and for comment counts. These partial correlations revealed significant positive associations between synchrony and awe (*pr* = 0.21, *p* < 0.001), admiration (*pr* = 0.22, *p* < 0.001), elevation (*pr* = 0.15, *p* < 0.001), and the overall self-transcendence measure (*pr* = 0.19, *p* < 0.001). We observed no significant associations for hope (*pr* = 0.07, *p* = 0.13) or for gratitude (*pr* = 0.03, *p* = 0.48).

We next examined if the relationship between synchrony and each of the self-transcendent scores was moderated by the search topic. Namely, we ran six analyses, one for each measure of self-transcendence, to examine if there were any interactions between synchrony and the search topic (dummy-coded) in predicting comments reflecting self-transcendent emotion. Total comment counts were employed as a covariate. PROCESS model 1 (Hayes, 2022) was employed to examine the significance of interactions as well as any associated simple effects. Across these analyses, the only significant interaction observed was for Gratitude, *F*(4, 478) = 2.99, *p* = 0.018, *R*^2^ change = 0.02. Simple slopes analysis showed that synchrony was unrelated to gratitude except for parades (*B* = 2.27, *SE* = 0.97, *p* = 0.02) and rituals (*B* = −2.34, *SE* = 1.06, *p* = 0.03). Overall, these results show that the topic of the videos had minimal influence on the relationship between synchrony and comments reflecting self-transcendent emotion.

Our next analyses examined whether each video characteristic moderated the relationship between synchrony and each of the self-transcendent scores. Specifically, we were interested in whether or not additional aspects of the video served to enhance or dampen the effects of synchrony on viewers’ comments reflecting self-transcendence. As in the previous analysis, we used PROCESS model 1 (Hayes, 2022) to examine the significance of interactions as well as any associated simple effects, controlling for the number of user comments. Table 2 reports these interaction coefficients as well as their standard errors.

Because so many tests were conducted, we are hesitant to devote too much attention to any single interaction because of the inflated risk of Type I errors. Because of the large number of tests conducted, these moderation analyses should be treated as exploratory and should be interpreted as pattern-finding rather than confirmatory hypothesis tests. Overall, synchrony in the video appears to be associated with higher rates of comments reflecting self-transcendent emotions when the participants in the video were perceived as wanting to be there and were friendly with one another. Likewise, the association between synchrony and self-transcendence appeared higher in dense and larger groups. Contrary to predictions, the depictions of anger, news, violence, and poor quality appeared inconsequential to the relationship between synchrony and comments reflecting self-transcendent emotion.

As in Study 1, our final analysis pertained to downstream effects regarding moral foundations. This analysis used synchrony scores as the IV and comments reflecting overall self-transcendence as the mediator. We opted not to include each individual type of self-transcendence, as these individual measures can contain overlapping words in the Self-Transcendent Emotions Dictionary (Ji et al., 2019). The focus of the video (dummy coded) was employed as a covariate.

**Figure 2:**
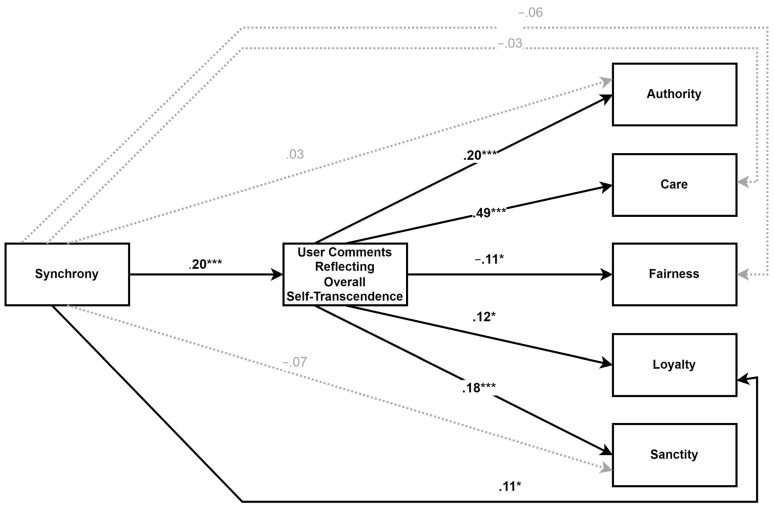
Study 2: Mediation Analysis of Indirect Effects of Synchrony. * *p* < 0.05. *** *p* < 0.001. *Note*. Path weights are standardized coefficients.

Figure 2 illustrates the results of this path analysis. As this figure shows, synchronization was a significant predictor of comments reflecting self-transcendent emotion (as demonstrated, too, in our analysis of variance). Further, comments reflecting self-transcendent emotion were positively associated with four of the five moral foundations: authority, care, loyalty, and sanctity. Curiously, comments reflecting self-transcendent emotion were negatively associated with the domain of fairness. Because we were particularly interested in the domain of care, we examined the bootstrapped difference between the path for care and the paths for every other domain. This analysis showed that the association between comments reflecting self-transcendent emotion and care was significantly higher than the paths for fairness, loyalty, and sanctity, and approached significance (*p* = 0.10) for the path for authority.

Additionally, the indirect association of synchrony was significant for the domains of authority (95% CI: [0.007, 0.086]); care (95% CI: [0.048, 0.146]); fairness (95% CI: [−0.050, −0.001]); and sanctity (95% CI: [0.016, 0.068]). The indirect association for loyalty fell short of significance (95% CI: [−0.012, 0.074]).

### 3.3. Brief Discussion

Our analysis of YouTube videos and their associated user comments showed that higher levels of synchrony were associated with higher levels of comments reflecting self-transcendence for awe, elevation, admiration, and for the overall measure of transcendence. This relationship appears to be most pronounced for videos featuring large and dense groups that appear to want to be together and are friendly with one another. In turn, videos with higher levels of comments reflecting self-transcendence were positively associated with higher levels of many moral domains, and particularly comments reflecting the domain of care.

## 4. General Discussion

The two studies reported in this paper employed different methodological approaches to examine the role of synchrony in social media videos on viewers’ responses related to self-transcendence and subsequent pro-social outcomes. Study 1 employed an experimental approach with a small subset of videos, whereas Study 2 broadened the sample of videos and employed user comments as indicators of viewer responses. Given the generally consistent findings across both studies, we believe that, together, these studies offer support for the idea that synchrony is a consequential aspect of video portrayals that are likely to elicit self-transcendent responses. Below we present an overall integration and interpretation of the findings of both studies.

This research on the novel concept of media synchrony sought to examine three questions. The first asked if media portrayals featuring synchrony elicit self-transcendent emotions (e.g., elevation, awe). Our results suggest that they do, albeit for awe and admiration and, to a lesser extent, elevation. The inconsistent relationship between synchrony and reports of elevation between Study 1 and Study 2 deserves our attention. In Study 1, there were no effects on elevation, whereas Study 2 revealed a significant, albeit small, positive relationship. We believe that this finding reflects that a wider array of videos and topics were employed in Study 2. In Study 1, synchronous videos of humans focused on group singing, with displays of moral virtue not present. In contrast, Study 2 included not only singing or dancing, but also rituals, parades, and rallies where we believe moral virtue may be more prominent.

Study 2 also showed no effects of synchrony on comments reflecting hope or gratitude. Perhaps emotions related to hope and gratitude situate the viewer as the recipient of some virtue, such as receiving relief, good news, or some offering. As such, future research may consider how synchrony may play a role in contexts where viewers may expect to receive some benefit from viewing, such as in the context of health-related videos or instructional tutorials that may provide information or support that is helpful to the viewer.

Our second question asked if there were downstream, indirect effects on prosocial outcomes, mediated by self-transcendence. Our path analyses suggested that there are, similar to a host of studies that have shown similar effects of elevating media. In Study 1, awe was associated with all of our measures of prosocial outcomes. In Study 2, we used a proxy of prosocial outcomes by measuring moral foundations. Given that the strongest outcome was associated with the moral foundation of care, we believe it mirrors the results obtained in Study 1. However, we also note that of the outcomes included in the moral dictionary, the foundation of fairness was negatively related to comments related to self-transcendence. Although we can only speculate about the reasons for this finding, an examination of fairness in the dictionary shows that it includes words such as “equity,” “payback,” “justice,” “revenge,” and “retaliation.” Such words imply some sort of inequality or circumstance that requires efforts to remediate the inequality. In contrast, synchrony is thought to generally reflect communal sharing and affiliation, so it may be at odds with the concept of fairness as it is operationalized in the dictionary. Ultimately, given that we employed this dictionary as a proxy for assessing prosocial outcomes, we look forward to the development of linguistic dictionaries that more directly assess prosocial outcomes like those employed in Study 1.

The final question asked what aspects of media can serve to increase or decrease the association of synchrony with viewers’ responses. In Study 1, we found that synchronous videos featuring humans or animals were more effective in eliciting awe than videos featuring objects. Study 2 expanded upon these moderators to suggest that comments reflecting self-transcendent emotion tended to be higher when synchronized groups appear to want to be there and are depicted in large, dense, and friendly groups.

The results of our study suggest that synchrony in media portrayals may be a consequential addition to the host of variables thought to increase self-transcendence. In some respects, this finding is not surprising given the number of social media channels that routinely display people dancing, singing, or moving in unison. Indeed, the popular channel “Playing for Change” (https://www.youtube.com/@PlayingForChange, accessed on 30 June 2026) appears to deliberately harness the power of synchrony for social good in their videos of geographically diverse musicians singing together. As their channel explains, “Playing For Change aims to create hope and inspiration for the future of our planet.” If synchrony functions to heighten feelings of affiliation and cohesion as suggested by “Playing for Change,” we can imagine a host of additional ways that it may be used in other contexts. For example, “positive news” outlets such as “Positive News” (https://www.positive.news/, accessed on 30 June 2026) or the “Good News Network” (https://www.goodnewsnetwork.org/, accessed on 30 June 2026) may consider including content that features synchrony more prominently. Likewise, synchrony may be an important design concept in persuasive messages used to encourage people to engage in greater cooperation for some common good such as battling pandemics or fighting climate change. Given that our empirical studies are admittedly exploratory, we believe that they can serve as an initial first step in exploring other ways that media and synchrony may function, including the aforementioned examples but also in additional contexts such as in co-viewing situations, in flash mobs, and message virality.

Of course, as with any research, these studies, too, come with limitations. First, in Study 1, we examined only a limited number of videos. We attempted to account for this by including two instantiations within each experimental condition. We also conducted our study using an online questionnaire. Although this approach likely introduced some noise into our study, we also believe that it more closely resembles actual media consumption than viewing in a lab.

In Study 2, we expanded upon the diversity of our video selections and used naturally occurring user comments as a proxy for viewer responses. However, the size of the sample of videos presented logistical barriers to using human coding to identify video characteristics, and we therefore employed AI agents as coders. Though we took careful steps in validating these AI ratings, we appreciate that AI falls short of the nuance of responses that human coders would likely have. For example, when compared to human ratings, AI agents rated the groups depicted as significantly larger and more dense. Indeed, ratings on a host of variables appeared stronger for the AI versus human agents, suggesting that they provide more extreme scores. We attempted to account for bias by averaging the scores for five AI agents for each measure rather than using a single AI rating, but given these limitations, these ratings should be interpreted as a proxy.

Finally, we are keenly aware of the problems associated with making causal claims using cross-sectional data. Although this approach is consistent with existing scholarship in media psychology, we look forward to more definitively establishing this causal connection by using longitudinal designs or by exploring ways that we may manipulate the mediator apart from that caused by the video stimulus materials.

With these limitations in mind, we hope that our research inspires interest in how the concept of synchrony may operate in media-related settings. From our vantage point, this novel approach opens up new possibilities of exploring how media displaying our shared movements and voices may ultimately lead us to greater affiliation and feelings of connection with others and with the planet.

## Figures and Tables

**Table 1:** Synchrony Scores.

	Search Synchrony		Across Both Synchrony Groups
Search Topic	No	Yes	
Dancing	6.50 _Ba_	6.94 _Aa_	6.72 _a_
	(0.12)	(0.11)	(0.09)
Drumming/Clapping	6.37 _Ba_	6.85 _Aab_	6.61 _a_
	(0.12)	(0.11)	(0.08)
Nature/Animals	4.95 _Bc_	6.61 _Ab_	5.78 _c_
	(0.11)	(0.12)	(0.08)
Parades/Marching/Walking	5.76 _Bb_	6.94 _Aa_	6.35 _b_
	(0.12)	(0.10)	(0.08)
Rituals/Ceremonies	5.80 _Bb_	6.80 _Aab_	6.30 _b_
	(0.13)	(0.13)	(0.09)
Across All Topics	5.88 _B_	6.83 _A_	
	(0.05)	(0.05)	

*Note*. Numbers are mean synchrony scores; numbers in parentheses are standard errors. Within rows, means with no uppercase subscript in common differ at *p* < 0.05 using LSD post hoc comparisons. Within columns, means with no lowercase subscript in common differ at *p* < 0.05 using LSD post hoc comparisons.

**Table 2:** Interaction Coefficients between Synchrony Scores and Video Characteristics in Predicting Comments Reflecting Self-Transcendent Emotion.

		Elev	Awe	Admir	Grat	Hope	Self-Tran
The Participants…							
	Wanted to Be There	0.12	0.63 ^+^	0.82 *	−0.26	0.21	0.65 ^+^
		(0.11)	(0.32)	(0.33)	(0.23)	(0.17)	(0.36)
	Enjoyed the Experience	0.07	0.40	0.62 ^+^	−0.19	0.26	0.54
		(0.12)	(0.35)	(0.35)	(0.25)	(0.18)	(0.39)
	Were Friendly with One Another	0.22 *	1.08 ***	1.34 ***	0.26	0.33 *	1.46 ***
		(0.10)	(0.29)	(0.30)	(0.21)	(0.15)	(0.32)
The Behaviors were…							
	Naturally Occurring	0.16	0.28	0.27	0.80 **	−0.10	0.64
		(0.12)	(0.37)	(0.38)	(0.26)	(0.19)	(0.41)
	Spontaneous	0.15	0.15	0.10	0.78 **	−0.16	0.46
		(0.12)	(0.37)	(0.38)	(0.26)	(0.19)	(0.41)
The Group depicted was…							
	Dense	0.18 ^+^	0.63 *	0.65 *	0.46 *	0.13	0.96 **
		(0.09)	(0.28)	(0.28)	(0.20)	(0.15)	(0.31)
	Large	0.17 *	0.61 *	0.65 *	0.43 *	0.10	0.89 **
		(0.08)	(0.25)	(0.25)	(0.18)	(0.13)	(0.28)
	Diverse	0.13	0.21	0.13	0.51 *	0.07	0.53^+^
		(0.09)	(0.28)	(0.29)	(0.20)	(0.14)	(0.32)
The Video…							
	Contained Violence	−0.09	0.07	−0.33	0.08	−0.40 *	−0.38
		(0.12)	(0.36)	(0.37)	(0.26)	(0.18)	(0.41)
	Contained Anger	0.08	0.79 ^+^	0.18	0.57 ^+^	−0.36	0.27
		(0.16)	(0.46)	(0.48)	(0.33)	(0.24)	(0.52)
	Was a News Story (% Yes)	0.25	1.35	1.07	1.69	0.69	2.62 ^+^
		(0.49)	(1.37)	(1.40)	(1.03)	(0.75)	(1.56)
	Was of Poor Video/Audio Quality	−0.05	−0.24	−0.62	0.79	−0.46	−0.18
		(0.33)	(0.96)	(0.99)	(0.69)	(0.50)	(1.08)

^+^ *p* < 0.10. * *p* < 0.05. ** *p* < 0.01. *** *p* < 0.001. *Note*. Numbers for each characteristic represent unstandardized interaction coefficients. Numbers in parentheses are standard errors. Elev = Elevation; Awe = Awe; Admir = Admiration; Grat = Gratitude; Hope = Hope; Tran = Overall Transcendence. Positive interaction coefficients reflect that the presence of the characteristic increased the relationship between synchrony and self-transcendence, whereas negative coefficients reflect that it decreased the relationship.

## Data Availability

Data for these studies are available upon request.

## AI-Use Statement

AI was employed in the collection of YouTube videos and in the coding of the videos. Perplexity AI (Perplexity Computer, Cloud-Native Agent) (https://www.perplexity.ai/, accessed 30 June 2026) was employed to generate a sample of YouTube URLs. Appendix B provides the prompts that were employed. Viewpoints AI, an AI-based research platform for designing and running studies with simulated personas, was employed to collect additional information concerning characteristics of these videos. These are powered by underlying LLMs, which are configured as part of the study. In the current study, the underlying model used was Gemini-2.5-flash. To assess the validity of using AI-Agent ratings as a proxy for human ratings, AI and human ratings were compared and are reported in Appendix C and Appendix D.

## Appendix A. Pretest of Videos Employed in Study 1

A pretest was conducted to inform the selection of videos used in Study 1. We invited participants from CloudResearch (https://www.cloudresearch.com/) to participate in an online study involving viewing and rating 1 of 24 videos. A total of 379 participants completed the study (61.5% female; median age = 39 years).

The 24 videos were approximately 2–3 min in length. Effort was taken to make sure that synchronous/asynchronous pairs were matched in terms of length, focus, and music. The videos involving humans included people singing “Have Ever Seen the Rain?,” people dancing, people doing acrobatics in a wind tunnel, and people singing “What’s Going On?”. The videos involving nature included underwater images of fish, birds flying, flowers, and sheep. The videos involving things included balls in motion, fountains, yard art, and origami.

After viewing the video, participants rated perceived synchrony on 7-point scales using seven items adapted from Bernieri (1988) (e.g., coordinated movement, behavioral similarity; Cronbach’s α = 0.91). Participants also rated the video in terms of elevation (moving, touching, meaningful; α = 0.90), awe (awe-inspiring, breathtaking, amazing; α = 0.91), and interest (enjoyable, interesting, boring (reversed), unpleasant (reversed); α = 0.85).

Based on these ratings, two video pairs were selected to represent people, nature, and objects. An analysis of variance was conducted on these six videos, with Table A1 displaying the means of ratings of perceived synchrony and evaluation. Based on these ratings, the two videos representing each category of people, nature, and things were collapsed. Subsequently, measures of elevation and awe were assessed using 2 (synchronous, not synchronous) × 3 (Category) analyses of variance (see Table A2). These analyses indicate that synchrony appears to elicit higher levels of awe for people and nature, and higher levels of elevation, but particularly for nature.

**Table A1:** Ratings of Perceived Synchrony and Interest.

	Perceived Synchrony		Interest	
Category and Topic	Synchrony Levels		Synchrony Levels	
	Low	High	Low	High
People				
Have You Ever Seen the Rain?	4.44	6.08	5.17	5.78
What’s Going On?	4.74	6.00	5.57	6.23
Nature				
Sheep	4.59	5.92	4.54	5.44 *
Fish	5.10	6.37	5.94	6.12
Objects				
Balls	4.75	5.87	5.27	5.95
Fountains	4.72	5.94	5.37	5.47

*Note*. A series of *t*-tests showed that for each video, synchronous videos were rated as significantly higher than asynchronous videos on perceived synchrony. In contrast, the only video rated significantly different on interest was the video featuring sheep. Among the synced videos, no significant difference was obtained for ratings of perceived synchrony [*F*(5, 88) = 0.83, *p* = 0.53, η_p_^2^ = 0.03] or interest [*F*(5, 88) = 1.31, *p* = 0.27, η_p_^2^ = 0.07]. Likewise, ratings of synchrony [*F*(5, 88) = 0.48, *p* = 0.70, η_p_^2^ = 0.03] and interest [*F*(5, 88) = 1.84, *p* = 0.11, η_p_^2^ = 0.10] did not differ among the asynchronous videos. * Differences between low and high synchrony levels, *p* < 0.05.

**Table A2:** Ratings of Awe and Elevation.

	Awe		Elevation	
	Synchrony Levels		Synchrony Levels	
	Low	High	Low	High
Category				
People	4.01 _A_	5.23 _B_	5.18 _Ab_	5.81 _Ab_
Nature	3.95 _A_	4.92 _B_	3.72 _Aa_	4.50 _Ba_
Things	3.95_A_	4.46 _A_	3.30 _Aa_	3.99 _Aa_

*Note*. Analyses of variance showed that for awe, there was a main effect of synchrony (*F*(1, 184) = 14.79, *p* < 0.001, η_p_^2^ = 0.07) but no main effect for category and no Synchrony X Category interaction. For elevation, there were main effects of synchrony (*F*(1, 184) = 9.60, *p* < 0.01, η_p_^2^ = 0.05) and category (*F*(1, 184) = 23.16, *p* < 0.001, η_p_^2^ = 0.20), but no Synchrony X Category interaction. Between columns, means with differing uppercase subscripts differ at *p* < 0.05 using least significant difference (LSD) contrasts (O’Keefe, 2003). Within columns, means with different lowercase subscripts differ at *p* < 0.05 using LSD contrasts.

## Appendix B. Prompts Used to Generate Video URLs

Parades/Marching/Walking

Synchronous Prompt: I would like you to retrieve a spreadsheet of YouTube videos and their meta data on the topic of parades, marching, or walking. The video should be between 30 and 420 s. The people in the video should be behaving in synchrony. The video should have at least 10 comments from users. You can retrieve up to 50 videos. There should be no duplicates, and no more than two videos should come from the same channel.

Non-Synchronous Prompt: Now I would like you to retrieve another spreadsheet of videos and their meta data using the same criteria and topic of parades, marching, or walking. This time, though, synchrony should not be as salient. People do not have to be in perfect synchrony.

Rituals/Ceremonies

Synchronous Prompt: I would like you to retrieve a spreadsheet of YouTube videos and their meta data on the topic of rituals or ceremonies. The video should be between 30 and 420 s. The people in the video should be behaving in synchrony. The video should have at least 10 comments from users. You can retrieve up to 50 videos. There should be no duplicates, and no more than two videos should come from the same channel.

Non-Synchronous Prompt: Now I would like you to retrieve another spreadsheet of videos and their meta data using the same criteria and topic of rituals or ceremonies. This time, though, synchrony should not be as salient. People do not have to be in perfect synchrony.

Drumming/Clapping

Synchronous Prompt: I would like you to retrieve a spreadsheet of YouTube videos and their meta data on the topic of people drumming or clapping. The video should be between 30 and 420 s. The people in the video should be behaving in synchrony. The video should have at least 10 comments from users. You can retrieve up to 50 videos. There should be no duplicates, and no more than two videos should come from the same channel.

Non-Synchronous Prompt: Now I would like you to retrieve another spreadsheet of videos and their meta data using the same criteria and topic of people drumming or clapping. This time, though, synchrony should not be as salient. People do not have to be in perfect synchrony.

Dancing

Synchronous Prompt: I would like you to retrieve a spreadsheet of YouTube videos and their meta data on the topic of dancing or performances similar to dancing. The video should be between 30 and 420 s. The people in the video should be behaving in synchrony. The video should have at least 10 comments from users. You can retrieve up to 50 videos. There should be no duplicates, and no more than two videos should come from the same channel.

Non-Synchronous Prompt: Now I would like you to retrieve another spreadsheet of videos and their meta data using the same criteria and topic of dancing or performances similar to dancing. This time, though, synchrony should not be as salient. People do not have to be in perfect synchrony.

Nature/Animals

Synchronous Prompt: I would like you to retrieve a spreadsheet of YouTube videos and their meta data on the topic of fish swimming in the ocean, birds, horses, sheep, gazelles, fireflies, or other animals. They should be in their natural environment. The video should be between 30 and 420 s. The animals should be synchronous in their behaviors. The video should have at least 10 comments from users. You can retrieve up to 50 videos. There should be no duplicates, and no more than two videos should come from the same channel.

Non-Synchronous Prompt: Now I would like you to retrieve another spreadsheet of videos and their meta data using the same criteria and topic fish swimming in the ocean, birds, horses, sheep, gazelles, fireflies, or other animals. They should be in their natural environment. This time, though, synchrony should not be as salient. The animals do not have to be in perfect synchrony.

## Appendix C. Test of Validity: Comparing Data from Study 1 with Responses from AI Agents

As our first test of the validity of the AI ratings of synchrony, we created a panel of 232 simulated personas that mirrored the demographic characteristics from our sample in Study 1. We then conducted a between-subjects experiment using the same videos and synchrony rating measures from study 1. Subsequently, we analyzed synchrony ratings in a 2 (Participant: Human, AI) × 2 (Synchrony: Yes, No) ANOVA. This analysis reveal significant main effects for both Synchrony, *F*(1, 460) = 202.97, *p* < 0.001, η_p_^2^ = 0.31, and Participant, *F*(1, 460) = 28.92, *p* < 0.001, η_p_^2^ = 0.06, as well as a Synchrony X Participant interaction, *F*(1, 460) = 31.85, *p* < 0.001, η_p_^2^ = 0.07. These effects reflected that synchrony scores were higher for the synchronized (*M* = 6.38, *SE* = 0.06) than unsynchronized (*M* = 5.13, *SE* = 0.06) videos, and also higher for the AI agents (*M* = 5.99, *SE* = 0.06) than the human participants (*M* = 5.52, *SE* = 0.06). The interaction occurred because both human and AI ratings were the same for the unsynchronized videos (human: *M* = 5.14, *SE* = 0.09; AI: *M* = 5.11, *SE* = 0.09), but synchrony ratings were higher among AI Agents (*M* = 6.86, *SE* = 0.06) than human participants (*M* = 5.89, *SE* = 0.09) for the synchronous videos. Nevertheless, both human and AI ratings were higher for the synchronized than unsynchronized videos. Hence, we concluded that the AI participants were able to detect synchronized movement in videos.

## Appendix D. Comparing Human and AI Ratings on a Subset of Videos

As a first means of testing the validity of these measures, 10 videos were randomly selected from our sample and were presented to human participants using Qualtrics and a sample from Prolific (https://www.prolific.com/). In a between-subjects design, 100 participants were randomly assigned to view and rate 1 of the 10 videos. The mean for each rating across the 10 participants for each video was computed and then correlated with the ratings from the AI agents.

For synchrony ratings, we averaged across the seven items to create an overall synchrony measure. Reliabilities were strong for both human participants (Cronbach’s α = 0.90) and for AI agents (Cronbach’s α = 0.81). The correlation between human and AI ratings across the 10 videos was strong, *r* = 0.57, *p* = 0.089. Though this did not reach traditional levels of statistical significance, the small number of videos examined clearly resulted in an underpowered test. An independent-samples *t*-test showed that AI ratings were significantly higher (*M* = 6.39, *SD* = 0.76) than were human ratings (*M* = 5.74, *SD* = 0.57), similar to the results obtained in the initial validity test.

For additional characteristics of the video, a series of correlations between human and AI ratings was conducted, as well as a series of independent-samples *t*-tests. Table A3 provides the means and standard deviations of each rating for human participants and AI Agents, the correlations between each rating, and t-statistics for the independent-samples *t*-tests. The correlations between the measures were all significant except for ratings that the video contained anger and video quality. However, for anger, scores on this measure were very low, suggesting that the truncation attenuated the correlation. For video quality, the correlation was not significant, but differences between the two scores were non-significant. The *t*-tests showed that the human and AI ratings were generally similar, with the exception of ratings of the density and size of the groups depicted and the % saying that the video was a news story. However, the magnitude of these differences was small, and the correlations for the variables were sizable, suggesting that AI does a reasonable job of reflecting human perceptions of the videos.

**Table A3:** *t*-tests and Correlations for Human vs. AI Ratings.

		Human Participants	AI Agents		
The Participants…		*M*	*M*	*r*	*t*
	Wanted to Be There	5.14	5.16	0.93 ***	−0.04
		(1.30)	(2.83)		
	Enjoyed the Experience	5.69	5.42	0.84 **	0.55
		(1.04)	(2.31)		
	Were Friendly with One Another	4.99	5.18	0.62 ^+^	−0.40
		(1.26)	(1.94)		
The Behaviors were…					
	Naturally Occurring	4.12	3.98	0.88 ***	0.33
		(2.11)	(2.75)		
	Spontaneous	3.69	3.96	0.94 ***	−0.77
		(1.99)	(2.7)		
The Group depicted was…					
	Dense	5.31	6.04	0.79 **	−2.98 *
		(0.88)	(1.25)		
	Large	5.03	6.20	0.81 **	−6.39 ***
		(0.86)	(0.97)		
	Diverse	2.96	3.76	0.77 **	−1.59
		(0.76)	(2.1)		
The Video…					
	Contained Violence	1.55	1.90	0.69 **	−0.78
		(0.86)	(1.9)		
	Contained Anger	1.22	1.10	0.02	1.19
		(0.22)	(0.25)		
	Was a News Story (% Yes)	67.70	88.00	0.78 **	−3.20 *
		(20.89)	(31.55)		
	Was of Poor Video/Audio Quality	5.47	3.78	0.26	1.86
		(1.09)	(2.94)		

*Note*. ^+^ *p* < 0.10. * *p* < 0.05. ** *p* < 0.01. *** *p* < 0.001. *Note*. Numbers in parentheses are standard deviations.
